# Diabetes Increases Risk of Cardiovascular Events in Patients Receiving Permanent Pacemaker: A Propensity Score-Matched Cohort Study

**DOI:** 10.1155/2022/6758297

**Published:** 2022-03-28

**Authors:** Huang-Chung Chen, Wen-Hao Liu, Chien-Hao Tseng, Yung-Lung Chen, Wei-Chieh Lee, Yen-Nan Fang, Shaur-Zheng Chong, Mien-Cheng Chen

**Affiliations:** Division of Cardiology, Department of Internal Medicine, Kaohsiung Chang Gung Memorial Hospital, College of Medicine, Chang Gung University, Taiwan

## Abstract

**Background:**

Type 2 diabetes was associated with a higher risk for permanent pacemaker (PPM) treatment. The difference in cardiovascular outcomes between patients with and without diabetes receiving PPM treatment remains unexplored.

**Method:**

Between January 2003 and December 2017, 1742 patients receiving naïve PPM treatment comprised this retrospective cohort study and were categorized into two groups by the diagnosis of diabetes: group with diabetes (*n* = 632, 36.3%) and group without diabetes (*n* = 1110, 63.7%). The primary outcome was cardiovascular events including heart failure (HF) hospitalization and acute myocardial infarction (AMI). The secondary outcomes of this study included pacemaker infection, pacing-induced cardiomyopathy, cerebrovascular accident, cardiovascular mortality, and all-cause mortality. Propensity score matching (PSM) was applied to reduce selection bias between the study groups.

**Result:**

During a mean follow-up period of 7.8 ± 4.8 years, 264 patients had a cardiovascular event. Before PSM, the incidence of cardiovascular events was higher in patients with diabetes compared to patients without diabetes (19.8% vs. 12.5%, *P* < 0.001), and the incidences of pacing-induced cardiomyopathy, cardiovascular mortality, and all-cause mortality were all higher in patients with diabetes compared to patients without diabetes. After PSM, the incidence of cardiovascular events was higher in patients with diabetes compared to patients without diabetes (18.8% vs. 12.3%, *P* = 0.015). The incidence of HF hospitalization was higher in patients with diabetes compared to patients without diabetes (15.3% vs. 10.2%, *P* = 0.037), whereas the incidence of AMI did not differ between the two groups. Moreover, after PSM, patients with diabetes had higher cumulative incidences of pacing-induced cardiomyopathy and all-cause mortality compared to patients without diabetes.

**Conclusions:**

The prevalence of diabetes was over one-third of naïve PPM recipients of this cohort, and diabetes increased the risk of cardiovascular events in PPM recipients, especially for HF hospitalization.

## 1. Introduction

Diabetes mellitus is a serious chronic disease with an imperative influence on the health of a human being in the world. Owing to the aging population, economic development, and change of lifestyle, the growth in global and regional prevalences of type 2 diabetes markedly increased [1–4]. The number of patients with type 2 diabetes had doubled during the past two decades, and half of people with diabetes are not even aware that they have diabetes [1, 4]. Diabetes is a well-known risk factor for cardiovascular events, such as acute myocardial infarction (AMI) and heart failure (HF) [5, 6]. Previous studies demonstrated that lethal tachyarrhythmia occurs commonly in patients with diabetes, possibly related to myocardial ischemia and sympathoadrenal activation in response to hypoglycemia [7, 8]. On the other hand, an association between bradyarrhythmia and diabetes has also been reported, which is possibly caused by microangiopathy and increased cholinergic sensitivity [9–11]. From a national diabetes registry study, Rautio et al. reported that type 2 diabetes was associated with a 1.6-fold higher risk for permanent pacemaker (PPM) treatment after adjustments for age, sex, and other factors [12]. However, the difference in cardiovascular outcomes between patients with and without type 2 diabetes receiving PPM treatment remains unexplored. Moreover, type 2 diabetes as an independent risk factor for cardiovascular events in pacemaker recipients remains unexplored. Accordingly, we conducted this retrospective cohort study to investigate and compare the clinical outcomes between patients with and without type 2 diabetes receiving PPM treatment after propensity score matching (PSM). Moreover, this study is also aimed at identifying whether type 2 diabetes increases risk of cardiovascular events in PPM recipients.

## 2. Methods

### 2.1. Study Cohort

**Figure 1:**
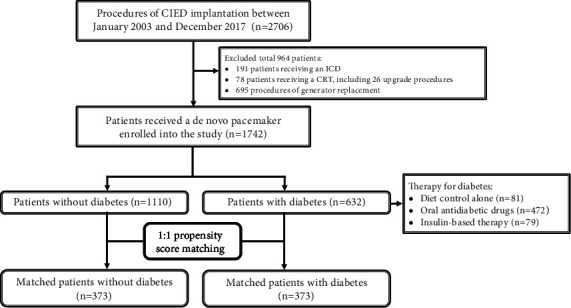
Flow chart of enrollment of patients receiving cardiac implantable electronic devices. CIED: cardiac implantable electronic devices; ICD: implantable cardioverter-defibrillator; CRT: cardiac resynchronization therapy.

This retrospective cohort study enrolled 2706 consecutive patients receiving cardiac implantable electronic devices implantation in our hospital between January 2003 and December 2017. A total of 964 patients, including 191 patients with implantable intracardiac defibrillators, 78 patients with cardiac resynchronization therapy, and 695 patients with replacement of generator, were excluded (Figure 1). His-Purkinje conduction system pacing was also excluded in this study because pacing leads for His-Purkinje conduction system pacing were not available in our institute between January 2003 and December 2017. Finally, 1742 patients receiving single ventricular or dual chamber PPMs comprised this retrospective cohort study population and were categorized into two groups by the presence or absence of diagnosis of type 2 diabetes at the time of PPM implantation: group with diabetes (*n* = 632, 36.3%) and group without diabetes (*n* = 1110, 63.7%) (Figure 1). The standard protocol for PPM implantation in our center had been described in our previous study [13], mainly right ventricular lead placed at the right ventricular outflow tract or high septum.

### 2.2. Definitions

Based on recommendations from the American Diabetes Association [14], diabetes was defined as prescription for oral antidiabetic drugs or insulin, or HbA1c ≥ 6.5% (48 mmol/mol), or fasting plasma glucose level ≥ 126 mg/dL (7.0 mmol/L), or a random plasma glucose ≥ 200 mg/dL (11.1 mmol/L) with classic symptoms of hyperglycemia or hyperglycemic crisis during hospitalization for PPM implantation. According to the guidelines of Kidney Disease: Improving Global Outcomes [15], microalbuminuria was defined as at least two positive results obtained within 1 year and was defined as an albumin-to-creatinine ratio of 30-300 mg/g (3-30 mg/mmol); macroalbuminuria was defined as an albumin-to-creatinine ratio ≥300 mg/g (>30 mg/mmol). Estimated glomerular filtration rate (eGFR) was estimated from the creatinine value and calculated using the Chronic Kidney Disease Epidemiology Collaboration equation [16]. Chronic kidney disease (CKD) was defined as eGFR lower than 60 mL/min/1.73 m^2^ without renal replacement therapy and end-stage renal disease as the need for peritoneal dialysis, hemodialysis, or renal transplantation. Hyperlipidemia was defined as total cholesterol ≥ 240 mg/dL, low-density lipoprotein ≥ 150 mg/dL, or triglyceride ≥ 200 mg/dL, or on lipid-lowering medications [17]. Valvular heart disease was defined as moderate to severe regurgitation or stenosis of aortic, mitral, or tricuspid valves. Cardiovascular surgery included coronary artery bypass graft and valvular surgery. Chronic lung disease was defined as a history of asthma, chronic obstructive pulmonary disease, or pulmonary fibrosis.

### 2.3. Clinical Outcomes

The primary outcome of this study was cardiovascular events of patients after PPM implantation. Cardiovascular events included hospitalization related to HF event of New York Heart Association functional class of III-IV, or AMI. The secondary outcomes of this study included pacemaker infection, pacing-induced cardiomyopathy, cerebrovascular accident, cardiovascular mortality, and all-cause mortality. Pacemaker infection was divided into major and minor infections according to clinical presentation and management. Major infection was defined as any presentation of (1) erosive wound, (2) bloodstream infection, (3) pacemaker-related endocarditis, or (4) need for surgical removal. Minor infection was defined as (1) the local inflammatory signs including erythema, warmth, fluctuance, or tenderness at the pocket sites, (2) presentation of any discharge, or (3) wound dehiscence [18]. Pacing-induced cardiomyopathy was defined as a ≥10% decrease of the baseline left ventricular ejection fraction (LVEF) with a resultant LVEF < 50%. Cerebrovascular accident was defined as an episode of transient ischemic attack, ischemic stroke, intracranial hemorrhage, or any incident finding by images, including brain computed tomography or magnetic resonance imaging after PPM implantation. Cardiovascular mortality was defined as death from AMI, HF, refractory ventricular arrhythmias, or cardiac arrest. After PPM implantation, patients were followed up monthly for the first three months and then every three to six months until clinical outcomes of interest, death, loss to follow up, or the latest date in the dataset (31 December, 2020), whichever came first.

### 2.4. Study Covariates

Baseline variables considered in the analyses included patient's age, sex, body mass index, and comorbidities associated with clinical outcomes including hypertension, hyperlipidemia, coronary artery disease, HF, atrial fibrillation, valvular heart disease, CKD, history of cardiovascular surgery, cancer, and chronic lung disease. The prescription for medication, such as beta-blocker, antihypertensive drugs, diuretic agents, and lipid-lowering agents, laboratory data including hemoglobin and serum creatinine, the indication and lead number of PPM, and baseline and pacing QRS duration were also obtained.

### 2.5. Statistical Analysis

Continuous variables are expressed as a mean ± standard deviation or percentages. The clinical characteristics of the study groups were compared using the independent *t*-test for continuous variables and Chi-square test or Fisher's exact test for categorical variables. PSM was applied to make the covariates balanced between the study groups. The propensity score was calculated using multivariable logistic regression where the study group was regressed on all of the covariates listed in Table 1, except eGFR, HbA1c, low-density lipoprotein, high-density lipoprotein, triglyceride, albuminuria, and preprocedural echocardiographic data. Using NCSS 10 Statistical Software (LLC, Kaysville, Utah, USA), the greedy method was used for matching at a 1 : 1 ratio between the study groups with a caliper width 0.2-fold of the standard deviation of the logit of the propensity score. The quality of matching was checked using the absolute value of standardized difference between the groups, where a value <0.1 was considered negligible difference [19]. The incidences of all clinical outcomes during long-term follow-up were expressed with Kaplan-Meier survival curves and compared by log-rank test. The significance of each variable in predicting all clinical outcomes was tested using the Cox proportional hazards model, analyzed with forward option. A two-sided *P* value < 0.05 was considered statistically significant. SPSS for Windows (version 22.0; SPSS Inc., Chicago, IL, USA) was used to perform the statistical analysis.

## 3. Results

### 3.1. Baseline Characteristics of the Study Patients with and without Type 2 Diabetes

Table 1 lists the clinical characteristics of the study patients before and after PSM. Before PSM, the mean age of the patient population was 73 ± 11 years and 48.6% of the study patients were male. There were 632 (36.3%) patients with diabetes, which were under diet control alone (12.8%), oral antidiabetic drugs (74.7%), or insulin-based therapy (12.5%), and 1110 (63.7%) patients without diabetes (Figure 1). The patients with diabetes had more patients with overweight and higher prevalence of hypertension, hyperlipidemia, coronary artery disease, CKD, and end-stage renal disease (all *P* < 0.001) compared to the patients without diabetes. Patients with diabetes also had a higher prevalence of history of HF (*P* = 0.033), atrial fibrillation (*P* = 0.007), and cerebrovascular accident (*P* = 0.028) compared to patients without diabetes. Patients with diabetes had more prescription for beta-blocker (*P* = 0.001), angiotensin-converting enzyme inhibitors/angiotensin receptor blocker (ACEi/ARB), diuretic agents, and statin (all *P* < 0.001). Patients with diabetes had higher serum creatinine, HbA1c, and triglyceride and a higher prevalence of albuminuria including microalbuminuria and macroalbuminuria (all *P* < 0.001) compared to patients without diabetes. Patients with diabetes had lower levels of hemoglobin, eGFR, low-density lipoprotein, and high-density lipoprotein (all *P* < 0.001) compared to patients without diabetes. Patients with diabetes had a higher prevalence of atrioventricular block (*P* = 0.001), larger number of PPM leads (*P* = 0.025), wider baseline and pacing QRS durations (*P* = 0.037 and *P* = 0.019, respectively), and higher percentage of right ventricular pacing (*P* < 0.001) compared to patients without diabetes. Patients with diabetes had larger preprocedural left atrial size (*P* = 0.010) and LV end-diastolic volume (*P* = 0.049) and lower preprocedural LVEF (*P* = 0.042) compared to patients without diabetes (Table 1).

In the study cohort after 1 : 1 PSM, 373 pairs with and without diabetes were analyzed. The baseline characteristics were balanced in the matched groups (Table 1). After PSM, the matched patients with diabetes still had lower low-density lipoprotein (*P* = 0.001) and high-density lipoprotein (*P* = 0.005) and higher triglyceride (*P* = 0.049) levels as well as higher prevalence of albuminuria (*P* < 0.001) compared to the matched patients without diabetes (Table 1).

### 3.2. Clinical Outcomes of the Study Patients with and without Type 2 Diabetes before and after PSM

During a mean follow-up period of 7.8 ± 4.8 years, before PSM, the incidence of cardiovascular events was higher in patients with diabetes compared to patients without diabetes (19.8% vs. 12.5%; hazard ratio (HR) = 2.06; 95% confidence interval (CI), 1.61-2.62; *P* < 0.001) (Table 2), and the incidences of pacing-induced cardiomyopathy (16.6% vs. 9.7%; HR = 2.24; 95% CI, 1.71-2.93; *P* < 0.001), cerebrovascular accident (13.1% vs. 12.7%; HR = 1.32; 95% CI, 1.00-1.73; *P* = 0.047), cardiovascular mortality (8.9% vs. 6.1%; HR = 1.81; 95% CI, 1.27-2.58; *P* = 0.001), and all-cause mortality (29.4% vs. 21.4%; HR = 1.75; 95% CI, 1.44-2.12; *P* < 0.001) were also higher in patients with diabetes compared to patients without diabetes (Table 2). The incidences of pacemaker infection including major and minor infections did not differ between the two groups (Table 2). After PSM, the incidence of cardiovascular events was still higher in patients with diabetes compared to patients without diabetes (18.8% vs. 12.3%; HR = 1.82; 95% CI, 1.25-2.63; *P* = 0.002) (Table 2). Patients with diabetes had a higher incidence of HF hospitalization compared to patients without diabetes (15.3% vs. 10.2%; HR = 1.78; 95% CI, 1.18-2.68; *P* = 0.006), whereas the incidence of AMI did not differ between the two groups (Table 2). After PSM, patients with diabetes had a higher incidence of pacing-induced cardiomyopathy (17.2% vs. 12.3%; HR = 1.62; 95% CI, 1.11-2.36; *P* = 0.013) and all-cause mortality (25.5% vs. 20.6%; HR = 1.41; 95% CI, 1.05-1.92; *P* = 0.023) compared to patients without diabetes (Table 2). However, after PSM, the incidences of pacemaker infection, cerebrovascular accident, and cardiovascular mortality did not differ between the two groups.

**Figure 2:**
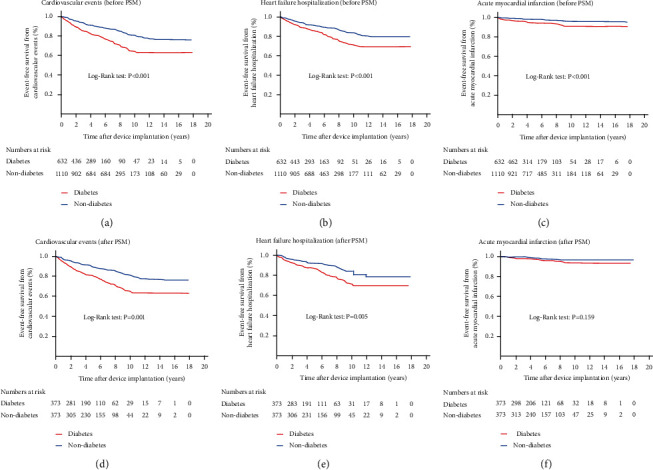
The Kaplan-Meier event-free survival curves of cardiovascular events (primary outcome) (a, d), heart failure hospitalization (b, e), and acute myocardial infarction (c, f) between the groups with and without diabetes before and after propensity score matching. PSM: propensity score matching.

**Figure 3:**
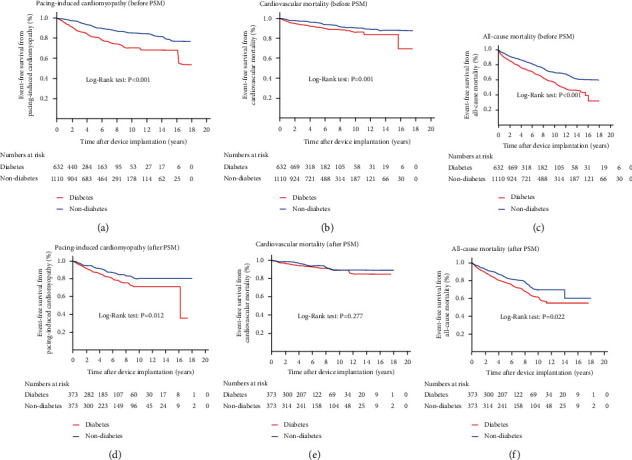
The Kaplan-Meier event-free survival curves of pacing-induced cardiomyopathy (a, d), cardiovascular mortality (b, e), and all-cause mortality (c, f) between the groups with and without diabetes before and after propensity score matching. PSM: propensity score matching.

The Kaplan–Meier curve analysis for cardiovascular events showed that patients with diabetes had a higher cumulative incidence of cardiovascular events compared to patients without diabetes before and after PSM (log-rank test, *P* < 0.001 and *P* = 0.001, respectively) (Figures 2(a) and 2(d)). Moreover, patients with diabetes had a higher cumulative incidence of HF hospitalization compared to patients without diabetes before and after PSM (log-rank test, *P* < 0.001 and *P* = 0.005, respectively) (Figures 2(b) and 2(e)). However, the cumulative incidence of AMI did not differ between the two groups before and after PSM (Figures 2(c) and 2(f)). The Kaplan–Meier curve analysis for pacing-induced cardiomyopathy showed that patients with diabetes had a higher cumulative incidence of pacing-induced cardiomyopathy compared to patients without diabetes before and after PSM (log-rank test, *P* < 0.001 and *P* = 0.012, respectively) (Figures 3(a) and 3(d)). Furthermore, patients with diabetes had a higher cumulative incidence of all-cause mortality compared to the patients without diabetes before and after PSM (log-rank test, *P* < 0.001 and *P* = 0.022, respectively) (Figures 3(c) and 3(f)). However, the cumulative incidence of cardiovascular mortality did not differ between the two groups after PSM (Figure 3(e)).

## 4. Discussion

In this cohort study, the prevalence of diabetes was 36.3%, over one-third of naïve PPM recipients. During a mean follow-up of 7.8 ± 4.8 years, after PSM, the incidences of cardiovascular events and HF hospitalization were significantly higher in patients with diabetes compared to patients without diabetes. Moreover, the cumulative incidences of cardiovascular events and HF hospitalization were significantly higher in the matched group with diabetes compared to the matched group without diabetes. Furthermore, patients with diabetes had a higher cumulative incidence of pacing-induced cardiomyopathy and all-cause mortality compared to patients without diabetes before and after PSM.

### 4.1. The Prevalence of Diabetes in Patients Receiving Pacemakers

The global prevalence of diabetes is rising from 8% in 1980 to 9.3% in 2019 and is estimated to be 10.9% by 2045, which may be attributable to population growth and ageing [1, 2]. In the Taiwanese population, the annual prevalence of diabetes increased significantly from 5.8% in 2000 to 8.3% in 2007, especially in the subgroup of men, age ≥ 80 years, and individuals residing in aging society areas [3]. In the elderly aged ≥65 years, around 15%-20% of people live with diabetes [1, 20]. In this study, PPM recipients were aged and the prevalence of diabetes was 36.3%, which was higher than general population [1–3, 20] and was compatible with previous data of PPM recipients [21, 22]. Moreover, similar to other reports [1–3, 20], the trend in the prevalence of diabetes in this study also increased from 28.8% (between 2003 and 2007) and 36.0% (between 2008 and 2012) to 41.4% (between 2013 and 2017).

Prior study reported that diabetes was possibly associated with sinus nodal dysfunction and cardiac conduction abnormalities [9–11, 23]. Movahed et al. reported that the incidence of complete atrioventricular block in patients with diabetes was 1.1%, which was 3-fold increased risk compared to patients without diabetes [11]. Patients with diabetes of this study had a higher prevalence of atrioventricular block compared to patients without diabetes (44.0% vs. 35.8%, *P* = 0.001) (Table 1), similar to other reports [10–12]. From a national diabetes registry study, Rautio et al. reported that diabetes increased 1.6-fold risk for implantation of PPM after adjustments for age, sex, and other factors [12]. Therefore, type 2 diabetes is a risk factor for PPM implantation and vigilant follow-up for bradyarrhythmia in patients with diabetes is necessary.

### 4.2. Heart Failure Hospitalization in Patients with Diabetes after Pacemaker Implantation

The prevalence of diabetes in HF patients is around 20%, and diabetes increased 1.74-fold risk and 1.95-fold risk of HF in men and women, respectively [6, 24]. In the Reduction of Atherothrombosis for Continued Health (REACH) Registry, diabetes was also associated with a 33% greater risk of HF hospitalization [25]. The reasons for increasing risk of HF in patients with diabetes included combined comorbidities, such as hypertension, acceleration of the development of coronary atherosclerosis, and diabetic cardiomyopathy, which was related to microangiopathy, metabolic factors, or myocardial fibrosis [24]. Moreover, a study using the National Readmission Database showed that the most common cause for readmission in PPM recipients was HF hospitalization [26]. Similar to general population, in this study of pacemaker recipients, we found that patients with diabetes had a higher risk for HF hospitalization compared to patients without diabetes (Table 2). Diabetic cardiomyopathy is characterized by diastolic relaxation abnormalities in its early stage and later systolic dysfunction [27]. The pathophysiological mechanisms of diabetic cardiomyopathy include systemic metabolic disorders, inappropriate activation of the renin–angiotensin–aldosterone system, subcellular component abnormalities, oxidative stress, inflammation and dysfunctional immune modulation, and finally, interstitial fibrosis of cardiac tissue, which contributed to substantial cardiac stiffness with diastolic dysfunction and later, systolic dysfunction [27]. Furthermore, diabetes is an important phenotype for HF with preserved LVEF and is also an independent predictor for HF hospitalization, despite under treatments of ACEi/ARB [28]. Interestingly, the study population in this study had preserved LVEF and the administration of ACEi/ARB was higher in patients with diabetes compared to patients without diabetes before PSM (Table 1). These findings deserve further investigations regarding angiotensin receptor-neprilysin inhibitor or sodium-glucose cotransporter 2 inhibitor in patients with diabetes with preserved LVEF for PPM implantation [29, 30].

Many studies have shown that right ventricular pacing is associated with HF hospitalization [31, 32]. Our prior study showed that right ventricular pacing QRS duration ≥ 163 milliseconds increased 3.5-fold risk of HF admission, and diabetes increased 2.7-fold risk of HF hospitalization [32]. Right ventricular pacing > 50% has been reported to be associated with an increased risk of HF hospitalization [31]. In this study, after PSM, the mean percentage of right ventricular pacing did not differ between patients with diabetes and patients without diabetes (Table 1). Moreover, the distribution of patients with right ventricular pacing > 40% was similar between patient with and without diabetes before and after PSM (Table 1). Of note, patients with diabetes had a higher cumulative incidence of pacing-induced cardiomyopathy compared to patients without diabetes before and after PSM, consequently, more HF hospitalization in patients with diabetes, and these findings were consistent with our prior study [32]. Recently, conduction system pacing, such as His-bundle pacing and left bundle branch pacing, has been reported to reduce HF hospitalization compared to right ventricular pacing [33, 34]. Our study was the first to show that diabetes was an independent predictor for cardiovascular events, including HF hospitalization, in patients after right ventricular PPM implantation, potentially related to more pacing-induced cardiomyopathy. Our findings provided the hypothesis for future studies of conduction system pacing in patients with diabetes who required PPM implantation.

### 4.3. Limitation

In this study, some potential limitations existed. First, although this was a retrospective single-center study, the sample size was large. Still, the potential bias inherent to nonrandomized investigations cannot be excluded. However, we performed PSM to minimize the bias between patients with and without diabetes. Second, the compliance period and dosage of prescription for beta-blocker, ACEi/ARB, diuretic agents, and statin during the follow-up period were not available in this study. Third, the duration of diagnosed diabetes before PPM implant was unknown. Finally, the preprocedural echocardiographic parameters of diastolic function by tissue Doppler or speckle-tracking imaging were not performed.

## 5. Conclusion

The prevalence of diabetes was over one-third of naïve PPM recipients of this cohort, and diabetes increased the risk of cardiovascular events in PPM recipients, especially for HF hospitalization.

## Figures and Tables

**Table 1:** Baseline characteristics of the study patients before and after propensity score matching.

	Before matching				After matching				
	Diabetes (*n* = 632; 36.3%)	Nondiabetes (*n* = 1110; 63.7%)	*P* value	SMD	Diabetes (*n* = 373)		Nondiabetes (*n* = 373)	*P* value	SMD
*Baseline characteristics*								
Age, (years)	73 ± 9	73 ± 12	0.080	0.080	74 ± 9	75 ± 11	0.232	0.088
Male	300 (47.5)	547 (49.3)	0.467	0.030	185 (49.6)	190 (50.9)	0.714	0.027
Body mass index, (kg/m^2^)	26 ± 4	24 ± 4	<0.001	N/A	25 ± 4	25 ± 4	0.046	N/A
Overweight (>30 kg/m^2^)	59 (9.3)	44 (4.0)	<0.001	0.187	22 (5.9)	26 (7.0)	0.551	0.044
Underweight (<20 kg/m^2^)	21 (3.3)	116 (10.5)	<0.001	0.248	17 (4.6)	21 (5.6)	0.505	0.049
Hypertension	524 (82.9)	716 (64.5)	<0.001	0.433	296 (79.4)	295 (79.1)	0.928	0.007
Hyperlipidemia	319 (50.5)	293 (26.4)	<0.001	0.567	160 (42.9)	163 (43.7)	0.825	0.016
Coronary artery disease	187 (29.6)	162 (14.6)	<0.001	0.368	79 (21.2)	74 (19.8)	0.650	0.033
Heart failure history	130 (20.6)	183 (16.5)	0.033	0.092	63 (16.9)	63 (16.9)	1.000	<0.001
Valvular heart disease†	27 (4.3)	68 (6.1)	0.101	0.081	19 (5.1)	21 (5.6)	0.745	0.024
Atrial fibrillation	219 (34.7)	457 (41.2)	0.007	0.188	143 (38.3)	147 (39.4)	0.764	0.022
Cerebrovascular accident	142 (22.5)	201 (18.1)	0.028	0.077	75 (20.1)	75 (20.1)	1.000	<0.001
Chronic kidney disease‡	291 (46.0)	396 (35.7)	<0.001	0.210	170 (45.6)	169 (45.3)	0.941	0.005
End-stage renal disease§	68 (10.8)	46 (4.1)	<0.001	0.254	20 (5.4)	20 (5.4)	1.000	<0.001
Chronic lung disease||	27 (4.3)	54 (4.9)	0.572	0.073	14 (3.8)	16 (4.3)	0.709	0.027
History of cardiovascular surgery	31 (4.9)	55 (5.0)	0.963	0.014	17 (4.6)	16 (4.3)	0.859	0.013
History of cancer	74 (11.7)	121 (10.9)	0.607	0.055	45 (12.1)	51 (13.7)	0.512	0.048
*Prescription for drugs*								
Beta-blocker	121 (19.1)	146 (13.2)	0.001	0.177	64 (17.2)	51 (13.7)	0.187	0.097
ACEi/ARB	370 (58.5)	506 (45.6)	<0.001	0.297	212 (56.8)	203 (54.4)	0.507	0.049
Diuretic agents	212 (33.5)	257 (23.2)	<0.001	0.265	113 (30.3)	114 (30.6)	0.937	0.006
Statin	193 (30.5)	152 (13.7)	<0.001	0.439	89 (23.9)	95 (25.5)	0.610	0.037
Diabetic therapy								
Diet control alone	81 (12.8)				59 (15.8)			
Oral antidiabetic drugs	472 (74.7)				282 (75.6)			
Insulin-based therapy	79 (12.5)				32 (8.6)			
*Laboratory data*								
Hemoglobin, (g/dL)	12.0 ± 1.9	12.8 ± 1.9	<0.001	0.409	12.3 ± 1.9	12.2 ± 1.9	0.754	0.023
Serum creatinine, (mg/dL)	2.0 ± 2.1	1.4 ± 1.7	<0.001	0.301	1.7 ± 1.8	1.6 ± 2.0	0.732	0.025
eGFR, (mL/min/1.73m^2^)	54 ± 30	67 ± 29	<0.001	N/A	58 ± 28	61 ± 29	0.171	N/A
HbA1c								
mmol/mol	54 ± 10	39 ± 3	<0.001	N/A	52 ± 8	39 ± 3	<0.001	N/A
%	7.1 ± 1.3	5.7 ± 0.4	<0.001	N/A	6.9 ± 1.1	5.7 ± 0.4	<0.001	N/A
LDL, (mg/dL)	90 ± 34	101 ± 34	<0.001	N/A	89 ± 33	101 ± 39	0.001	N/A
HDL, (mg/dL)	47 ± 13	53 ± 16	<0.001	N/A	48 ± 13	52 ± 15	0.005	N/A
Triglyceride, (mg/dL)	128 ± 87	103 ± 55	<0.001	N/A	124 ± 99	109 ± 59	0.049	N/A
Albuminuria, (mg/g)	141 (22.3)	70 (6.3)	<0.001	N/A	89 (23.9)	36 (9.7)	<0.001	N/A
Microalbuminuria	91 (14.4)	53 (4.8)	<0.001	N/A	58 (15.5)	25 (6.7)	<0.001	N/A
Macroalbuminuria	50 (7.9)	17 (1.5)	<0.001	N/A	31 (8.3)	11 (2.9)	0.001	N/A
*Electrocardiographic and pacemaker data*								
Patients with atrioventricular block	278 (44.0)	397 (35.8)	0.001	0.282	154 (41.4)	156 (41.8)	0.882	0.011
Number of pacemaker lead	1.9 ± 3.8	1.8 ± 3.8	0.025	0.107	1.9 ± 0.3	1.9 ± 0.3	0.661	0.034
Baseline QRS duration (ms)	103 ± 25	101 ± 24	0.037	0.127	103 ± 24	103 ± 25	0.928	0.007
Pacing QRS duration (ms)	167 ± 19	164 ± 19	0.019	0.154	165 ± 17	164 ± 18	0.180	0.098
Percentage of right ventricular pacing (%)	64 ± 42 (range: 0-100)	52 ± 44 (range: 0-100)	<0.001	N/A	62 ± 42 (range: 0-100)	54 ± 42 (range: 0-100)	0.075	N/A
Percentage with right ventricular pacing >40% (%)	146 (23.1)	267 (24.1)	0.677	N/A	95 (25.5)	89 (23.9)	0.610	N/A
*Pre-procedural echocardiographic data*								
LA size, (mm)	39 ± 7	38 ± 8	0.010	N/A	39 ± 7	39 ± 8	0.895	N/A
LVEDV, (ml)	114 ± 35	110 ± 38	0.049	N/A	114 ± 33	113 ± 39	0.676	N/A
LVESV, (ml)	39 ± 23	37 ± 38	0.065	N/A	38 ± 21	38 ± 24	0.898	N/A
LVEF, (%)	67 ± 12	68 ± 11	0.042	N/A	68 ± 11	67 ± 12	0.876	N/A

^∗^Data are presented as mean ± SD or number (%) of patients. ^†^Defined as moderate to severe regurgitation or stenosis of aortic, mitral, or tricuspid valves. ^‡^Defined as eGFR lower than 60 mL/min/1.73 m^2^ without renal replacement therapy. ^§^Defined as the need for peritoneal dialysis, hemodialysis, or renal transplantation. ^||^Defined as the history of asthma, or chronic obstructive pulmonary disease, or pulmonary fibrosis. ACEi/ARB: angiotensin-converting enzyme inhibitors/angiotensin receptor blocker; eGFR: estimated glomerular filtration rate; HbA1c: hemoglobin A1c; HDL: high-density lipoprotein; LA: left atrium; LDL: low-density lipoprotein; LVEDV: left ventricular end-diastolic volume; LVEF: left ventricular ejection fraction; LVESV: left ventricular end-systolic volume; N/A: not applicable; SMD: standardized mean difference.

**Table 2:** Clinical outcomes of the patients with and without diabetes and univariate Cox regression analysis for hazard ratio of diabetes vs. nondiabetes for all outcomes during a nearly 8-year follow-up period.

	Before matching				After matching			
	Diabetes (*n* = 632)	Nondiabetes (*n* = 1110)	HR (95% CI)	*P* value	Diabetes (*n* = 373)	Nondiabetes (*n* = 373)	HR (95% CI)	*P* value
Primary outcome								
Cardiovascular events	125 (19.8)	139 (12.5)	2.06 (1.61-2.62)	<0.001	70 (18.8)	46 (12.3)	1.82 (1.25-2.63)	0.002
HF hospitalization	94 (14.9)	112 (10.1)	1.91 (1.45-2.52)	<0.001	57 (15.3)	38 (10.2)	1.78 (1.18-2.68)	0.006
AMI	31 (4.9)	27 (2.4)	2.47 (1.47-4.15)	0.001	13 (3.5)	8 (2.1)	1.87 (0.77-4.51)	0.165
Secondary outcomes								
Pacemaker infection	16 (2.5)	28 (2.5)	1.00 (0.54-1.87)	0.991	14 (3.8)	8 (2.1)	1.78 (0.74-4.29)	0.200
Major infection	3 (0.5)	4 (0.4)	1.32 (0.29-5.91)	0.718	2 (0.5)	1 (0.3)	2.01 (0.18-22.21)	0.571
Minor infection	13 (2.1)	24 (2.2)	0.95 (0.48-1.88)	0.884	12 (3.2)	7 (1.9)	1.74 (0.68-4.47)	0.251
PICM	105 (16.6)	108 (9.7)	2.24 (1.71-2.93)	<0.001	64 (17.2)	46 (12.3)	1.62 (1.11-2.36)	0.013
Cerebrovascular accident	83 (13.1)	141 (12.7)	1.32 (1.00-1.73)	0.047	56 (15.0)	49 (13.1)	1.33 (0.91-1.95)	0.146
Cardiovascular mortality	56 (8.9)	68 (6.1)	1.81 (1.27-2.58)	0.001	25 (6.7)	21 (5.6)	1.38 (0.77-2.46)	0.279
All-cause mortality	186 (29.4)	237 (21.4)	1.75 (1.44-2.12)	<0.001	95 (25.5)	77 (20.6)	1.41 (1.05-1.92)	0.023

^∗^Data are presented as number (%) of patients. AMI: acute myocardial infarction; CI: confidence interval; HF: heart failure; HR: hazard ratio; PICM: pacing-induced cardiomyopathy.

## Data Availability

The retrospective data used to support the findings of this study are available from the corresponding author upon request.
